# The phenotypical expression of an European inherited TTR amyloidosis in Brazil

**DOI:** 10.1186/1750-1172-10-S1-O7

**Published:** 2015-11-02

**Authors:** Márcia Waddington Cruz, Débora Foguel, Amanda Berensztejn, Roberto Pedrosa, Priscila Ferreira da Silva

**Affiliations:** 1 Federal University of Rio de Janeiro, CEPARM. Neurology, 21941913, Rio de Janeiro, Brazil; 2 Federal University of Rio de Janeiro, CEPARM. Instituto de Bioquímica Médica, 21941913, Rio de Janeiro, Brazil; 3 Federal University of Rio de Janeiro, CEPARM. Cardiology, 21941913, Rio de Janeiro, Brazil

## Background

Brazil is a country of Portuguese colonization with massive numbers of immigrants and Portuguese descendants (25 millions). At least half of all Brazilian Y chromosomes are from Portuguese origin. Nevertheless, this population suffered miscegenation with native Indians and African descendants. African descendants Brazilians have 48% of non-African genes, probably from Portuguese ascendance. In a previous work from our center a common haplotype was demonstrated in Portuguese and Brazilian patients from 22 families and the calculation of the most recent common ancestor in 13 families demonstrated that it has occurred at 26 past generations about 650 years ago hence before the time of Brazil's discovery (1500) [1]. The objective of this work is to characterize demographic and clinical aspects of Brazilian patients presenting with ATTR in light of the clear Portuguese origin of the cases in a background of miscegenation and heterogeneity of the population.

## Methods

Baseline clinical and demographics aspects of Brazilian patients included into THAOS (Transthyretin Amyloidosis Outcomes Survey) patient registry were extracted from 2007 to January 2015 in a total of 148 patients (68 women and 80 men).

## Results

Val30MetTTR mutation was found in 91.9% of the cases. Other mutations included Ile107Val, Val122Ile, Ala19Asp and Gly53Glu. The mean age at onset of disease was 37 years for men and 35 years for women. Mean time from onset of symptoms to diagnosis was 5.9 years (median 3.3 years). 93.9% informed a family history with more than 90% of Portuguese origin and 69% with aspects from Caucasian ethnicity. In 23% of the cases, the diagnosis in family members was based on clinical suspicion only. Amyloid deposit was found in 80% of the biopsies performed (34% salivary gland, 38% nerve and 3.7 cardiac). Misdiagnosis was noticed in 27.4% of the cases (26% of Val30Met mutations and 37.5% of non Val30 Met mutations cases) with CIDP being the most common. 25% of the patients took more than 1 year to have their correct diagnosis. From 117 symptomatic patients 79.5% presented with motor neuropathy (78% Val30 Met and 87.5% non-Val30Met); 85.5% presented with sensory neuropathy (85% Val30 Met and 87.5% non-Val30Met); 93% presented with autonomic neuropathy (93.6% Val30 Met and 87.5% non-Val30Met); 80.3% presented with gastrointestinal complaints. Unintentional weight loss was present in 50% of the cases. 80.6% of early onset cases presented with a motor neuropathy and 78.3% of late onset. Corresponding numbers for sensory neuropathy were 87% and 82.6%; and 94.6% and 87% for autonomic neuropathy. Cardiac disorders were noted in 35.9% of the cases (33.9% Val30 Met and 62.5% non-Val30Met 33.9%) (NYHA 1 or > in 3% of patients with early onset and 17.4% of late onset). ECG abnormalities were found in 56% of the cases (71% being conduction abnormalities). Left ventricular septum > 10 mm was seen in only 25% of the cases.

## Conclusion

The population was mostly characterized by early onset TTR Val30Met neuropathic phenotype presentation, with several cases also featuring some degree of cardiac disease, very similar to cases from endemic regions of Portugal even after several generations from the original immigration and a very important mixed racial population.
